# Pharmacokinetics and Pharmacogenetics of Cyclophosphamide in a Neonate and Infant Childhood Cancer Patient Population

**DOI:** 10.3390/ph14030272

**Published:** 2021-03-16

**Authors:** Shelby Barnett, Julie Errington, Julieann Sludden, David Jamieson, Vianney Poinsignon, Angelo Paci, Gareth J. Veal

**Affiliations:** 1 Newcastle University Centre for Cancer, Newcastle University, Newcastle upon Tyne NE2 4HH, UK; shelby.barnett@ncl.ac.uk (S.B.); julie.errington@ncl.ac.uk (J.E.); julieann.sludden@ncl.ac.uk (J.S.); david.jamieson@ncl.ac.uk (D.J.); 2 Department of Pharmacology and Drug Analysis, Gustave Roussy Cancer Campus Grand Paris, Université Paris-Sud, 94805 Villejuif, France; vianney.poinsignon@gmail.com (V.P.); angelo.paci@gustaveroussy.fr (A.P.)

**Keywords:** cyclophosphamide, pharmacokinetics, paediatrics, neonates, infants, cancer

## Abstract

Infants and young children represent an important but much understudied childhood cancer patient population. The pharmacokinetics and pharmacogenetics of the widely used anticancer prodrug cyclophosphamide were investigated in children <2 years of age. Concentrations of cyclophosphamide and selected metabolites were determined in patients administered cyclophosphamide at doses ranging from 100–1500 mg/m^2^ (5–75 mg/kg), with various infusion times as determined by the standard treatment regimen that each patient was receiving. Polymorphisms in genes including CYP2B6 and CYP2C19 were investigated. Data generated for cyclophosphamide were analysed using a previously published population pharmacokinetic model. Cyclophosphamide pharmacokinetics was assessed in 111 samples obtained from 25 patients ranging from 4–23 months of age. The average cyclophosphamide clearance for the patients was 46.6 mL/min/m^2^ (ranging from 9.4–153 mL/min/m^2^), with marked inter-patient variability observed (CV 41%). No significant differences in cyclophosphamide clearance or exposure (AUC) were observed between patient groups as separated by age or body weight. However, marked differences in drug clearance and metabolism were noted between the current data in children <2 years of age and recently published results from a comparable study conducted by our group in older children, which reported significantly lower cyclophosphamide clearance values and metabolite exposures using the same population pharmacokinetic model for analysis. Whilst this study demonstrates no significant differences in cyclophosphamide clearance in patients <2 years, it highlights large differences in dosing protocols across tumour types. Furthermore, the study suggests marked differences in cyclophosphamide clearance in children less than two years of age as compared to older patients.

## 1. Introduction

**Figure 1:**
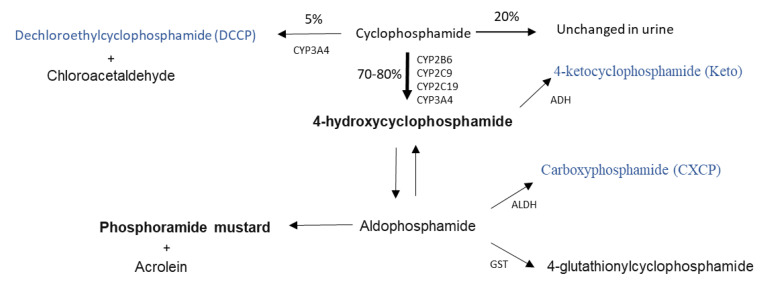
Cyclophosphamide metabolic pathway (adapted from Jonge et al., 2005 [7]). Active metabolites are bold, metabolites measured in this study are blue. CYP; Cytochrome P450, ADH; Alcohol dehydrogenase, ALDH; aldehyde dehydrogenase, GST; Glutathione-S-transferase.

The anticancer drug cyclophosphamide is widely used for the treatment of numerous childhood cancer malignancies, including neonates and infants, and is likely to remain an important chemotherapeutic for the years to come [1,2]. Cyclophosphamide, like other oxazaphosphorines, is a prodrug and is required to undergo a relatively complex pathway of metabolism to generate its active alkylating form phosphoramide mustard via 4-hydroxycyclophosphamide (Figure 1), which can then target DNA replication in tumour cells, alongside the formation of numerous inactive or toxic metabolites [2].

While many studies have been published investigating the pharmacokinetics and pharmacogenetics of cyclophosphamide in both adult and childhood cancer settings, there remains a dearth of information relating to the clinical pharmacology of cyclophosphamide in neonates and infants [3,4]. This leads to uncertainties as to the most appropriate dosing regimens to use in these patient populations, which currently vary between tumour type and clinical treatment protocols, and are commonly supported by relatively limited scientific rationale. For example, cyclophosphamide dose may be based on mg/kg up to 1 year of age or up to a weight of 10 kg in one clinical study protocol, as compared to body surface area-based dosing in another. In addition, commonly implemented dose reductions of 25–50% are applied inconsistently between studies and with different body weight or age cut-off points for a wide range of drugs, as previously discussed [5,6]. These factors can lead to substantial differences in the dose of drug administered to neonates and infants as compared to older children, particularly for infants marginally above or below defined dosing cut-offs.

Although such differences and inconsistencies in dosing regimens for neonates and infants are not uncommon for chemotherapeutics used in paediatric oncology, it may be particularly important for cyclophosphamide due to its complex metabolism and potential maturational differences in the ontogeny of key enzymes involved in its activation. Indeed, a recently published study from the US has highlighted a relationship between exposure to the active metabolite 4-hydroxycyclophosphamide and toxicity, and proposed a reduced cyclophosphamide dosage for young infants who experienced higher exposures, as compared to older children in a brain tumour setting [8].

The current study was designed to investigate the pharmacokinetics (plasma concentrations and observed level of inter-patient variation in drug exposure) and pharmacogenetics of cyclophosphamide in children <2 years of age at diagnosis. Patients recruited to the study received cyclophosphamide according to the defined dosing regimen for the clinical trial on which they were being treated. Patients were recruited into each of three defined age groups (0–6 months, 7–12 months, and 13–24 months), in order to investigate potential differences in neonate and infant populations where very limited clinical pharmacology data currently exist. The data generated were compared to previously published data generated by our group, using the same population pharmacokinetic model in older children receiving cyclophosphamide.

## 2. Results

### 2.1. Patient Characteristics and Treatment

Twenty-five patients below 2 years of age receiving cyclophosphamide as part of their standard clinical treatment were recruited to the study across eight UK centres, over a six-year period. Patient characteristics are summarized in Table 1. Recruitment to this study was not tumour specific, but dependent on the criteria mentioned previously. Therefore, patients with a wide variety of tumour types were recruited to the study. As patients were treated according to study protocols or guidelines for several different tumour types, this translated to a large range of chemotherapy dosing regimens being utilised, as highlighted in Table S1.

### 2.2. Cyclophosphamide Pharmacokinetics

The cyclophosphamide population pharmacokinetic analysis using data from 25 patients, with a total of 111 samples available, was performed using NONMEM 7.2. As previously described [9,10], creatinine and CYP2B6*6 genotype were used as covariates because of their relationship with cyclophosphamide clearance. The two-compartment model included random effects on CL, V1, Q and V2 allowing for correlation between CL and V1. Allometric scaling was used to allow for differences in body size; population parameters are therefore scaled to a standard body surface area of 1.4 m^2^. Parametrization of both CL and V1 are presented in Table S3. Average parameters (geometric mean) were CL 46.6 ± 19.2 mL/min/m^2^, V1 9.7 ± 2.4 L/m^2^, Q 0.28 ± 0.15 L/h/m^2^ and V2 3.6 ± 4.10^−^^5^ L/m^2^. The parameter and inter-individual estimates adequately describe the observed data, as shown by the diagnostic plots in Figure S1. The 1000 simulations carried out to provide VPC resulted in 86,000 simulated observations, among which 11.6% were outside the 90% prediction intervals (Figure S1).

**Figure 2:**
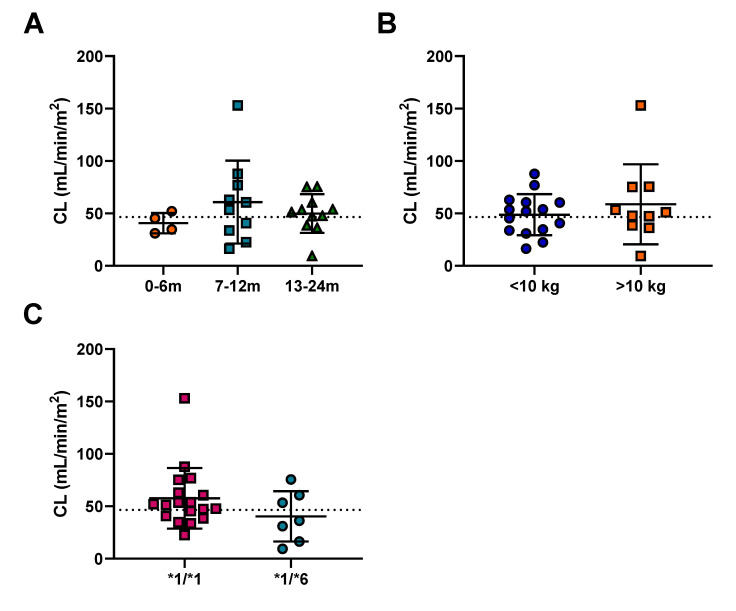
The effect of (**A**) age, (**B**) weight and (**C**) CYP2B6 genotype on cyclophosphamide clearance. The error bars represent standard deviation, the dashed line indicates the mean cyclophosphamide clearance of 52.8 mL/min/m^2^.

Cyclophosphamide pharmacokinetics was assessed in 25 patients ranging from 4 to 23 months of age. Given the differences in size of the patients, clearance was normalized to surface area. The average cyclophosphamide clearance for the patients was 46.6 mL/min/m^2^ (ranging from 9.4–153 mL/min/m^2^), with marked inter-patient variability observed (CV 41%). However, no significant differences in cyclophosphamide clearance between patient groups as separated by age or body weight were observed (Figure 2). In addition, given the large difference in dosing protocols, AUC was normalized to the dose each patient received. The average AUC for these patients was 0.045 (mg/mL.min)/dose (Table 2). Exposure to cyclophosphamide was again highly variable between patients (CV 62%) (Table 2), despite accounting for the different dosing regimens utilized (Table S1). Although there were no significant differences in normalized exposure between age groups and body weight (Figure S2A,C), there was a trend (*p* = 0.09) towards decreased exposure in patients over 10 kg (Figure S2C). This was not apparent from the raw AUC data (Figure S2D) and is likely an artefact of normalization, due to a weak positive correlation between weight and dose (R^2^ = 0.292).

**Figure 3:**
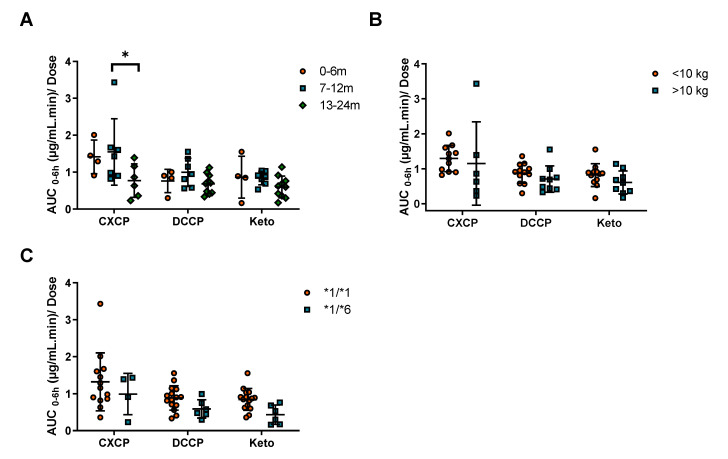
The impact of (**A**) age, (**B**) weight and (**C**) CYP2B6 genotype, on cyclophosphamide dose normalized exposure (AUC_0–6h_) of cyclophosphamide metabolites. * Indicates a significance level of *p* < 0.05.

The pharmacokinetics of cyclophosphamide metabolites were also investigated in the current study. However, due to insufficient numbers of samples, this analysis could only be conducted in 20 out of the 25 patients. In this instance, the AUC of metabolites CXCP, DCCP and Keto were calculated from 0–6 h for each patient. The AUC was then normalized for the dose of the parent drug for each subject. No significant differences in exposure were observed between patients above or below 10 kg for any of the metabolites (Figure 3B). In contrast, a significant decrease in CXCP exposure was observed for patients >13 months of age compared to patients 7–12 months (Figure 3A). No significant differences were observed for the other metabolites across the age ranges (Figure 3A).

**Figure 4:**
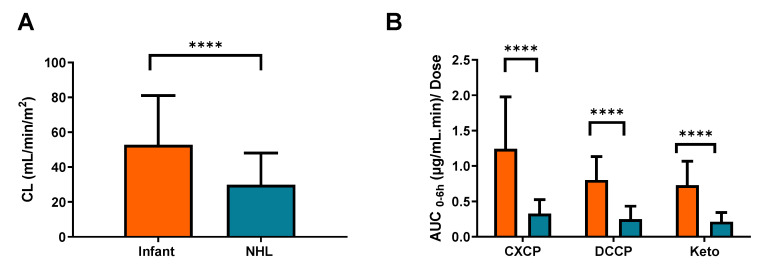
Observed differences between (**A**) cyclophosphamide clearance and (**B**) metabolite AUC_0–6h_ in patients from the current infant study (Orange) and a recently published study focusing on older patients with NHL (Blue). The error bars represent standard deviation, **** indicates a significance level of *p* < 0.0001.

The data generated were compared with previous data generated using the same analytical techniques and analysed using the same population pharmacokinetic model in a population of 49 childhood cancer population aged 3–19 years [10]. These previously published data in older children showed an average cyclophosphamide clearance of 1.83 ± 1.07 L/h/m^2^ (30.5 ± 17.8 mL/min/m^2^), as compared to 46.6 mL/min/m^2^ in children <2 years in the current study. Despite the wide level of variability in each of these patient populations, cyclophosphamide clearance values were markedly higher in the younger patient group in the current study (*p* < 0.0001) as shown in Figure 4A. Significant differences were also observed in exposure to cyclophosphamide metabolites, in terms of a markedly lower AUC normalized to cyclophosphamide dose administered in older children (Figure 4B), supporting the observation of a decrease in CXCP exposure in patients >13 months as compared to 7–12 months described above.

### 2.3. Pharmacogenetics

Pharmacogenetic analysis was performed to identify patients with relevant SNPs associated cyclophosphamide pharmacokinetics. From this analysis the numbers of patients for the CYP genotypes are as follows: *1/*1 (19), *1/*5 (5) and *5/*5 (1) for CYP2B6*5; 1/*1 (18) and *1/*6 (7) for CYP2B6*6, 1/*1 (20) and *1/*2 (5) for CYP2C19*2. The CYP2B6*6 SNP was identified as an important covariate for describing the population pharmacokinetics of cyclophosphamide. There was a trend towards increased exposure and decreased clearance for the *1/*6 genotype (Figure 2C). This change in clearance was 30%, however it was not statistically significant due to the limited number of patients recruited to the study. In addition, no significant difference between CYP2B6 genotypes was observed for cyclophosphamide exposure (Figure S2B), although the mean AUC/D for the *1/*6 genotype was 85% higher than the wild-type. In contrast, trends towards decreased exposure were observed for all cyclophosphamide metabolites (Figure 3C). However, in each case these differences were not significant, likely due to the small number of infants in the *1/*6 group (*n* = 7). Combining the results observed in the current study with data from the previously published study in older children, a significant reduction in clearance associated with the *1/*6 genotype is observed (Figure S3).

### 2.4. Toxicity and Response

Patient follow up was assessed for 12 months following the initial pharmacokinetic sampling. Out of the 25 patients studied, 22 patients (88%) were alive after 12 months, with 17 patients (68%) alive with no disease. The three patients that died during the one year follow up were due to disease progression (*n* = 2) and relapse (*n* = 1). Following cyclophosphamide treatment, any related toxicities grade 3 or above were recorded (Table S2). Only four out of the 25 infants (16%) had no toxicities reported following cyclophosphamide treatment. The most common toxicities observed were haematological, with decreased neutrophil and haemoglobin count occurring in approximately half of the patients (12 in 25). In addition, reduced WBC count (10 in 25), reduced platelet count (three in 25), fever (four in 25), infection (four in 25) and diarrhoea (two in 25) were reported in patients but to a lesser frequency. Other toxicities were reported however they were each only observed in a single patient and are described in Table S2. No trends were associated between increased exposure of metabolite or parent and any of the toxicities mentioned above. Patients were receiving a variety of co-medications, including other chemotherapeutic agents depending on their chemotherapy regimen. Therefore, attributing these toxicities solely to cyclophosphamide administration was not possible.

## 3. Discussion

As limited data currently exist relating to the pharmacokinetics of cyclophosphamide in neonates and infants, we have assessed the pharmacokinetics of cyclophosphamide and its metabolites in 25 patients under the age of 24 months. The collection of pharmacokinetic data for a specific drug in neonate and infant patient populations is a challenging initiative, as highlighted by the length of time taken to complete the current study. However, the availability of clinical pharmacology data in this patient population has the potential to provide valuable insights which may help to guide future dosing regimens [11,12]. This is particularly pertinent in the case of cyclophosphamide, a commonly use anticancer prodrug which requires metabolic activation into its active metabolites.

With patients categorized by age (0–6, 7–12, and 13–24 months) or weight (above and below 10 kg), there were no significant differences in cyclophosphamide clearance observed between patient groups. This was due to large inter-patient variability (CV 41%) in clearance for these infants and neonates, consistent with other drugs used in this age group such as carboplatin and etoposide [13]. This would suggest that, based on a purely pharmacological rationale, there is no clear evidence for cyclophosphamide dose reductions, when comparing younger patients above and below the commonly used age or body weight cut-off values for reduced dosing (commonly one year of age or 10 kg body weight). Across the patient cohort studied a wide range of cyclophosphamide doses (100–1500 mg/m^2^) were administered, as a result of differences in the dosing protocols being followed. Although, there were differences in dosing across protocols, there were no significant differences in clearance or exposure observed between tumour types (Figure S4). Although there was a trend towards lower exposures in the neuroblastoma group, for those dosed at 5–10 mg/kg/day, these patients are dosed over five consecutive days and their cumulative exposure would therefore be comparable to the other dosing regimens studied.

While in the current study no significant differences in cyclophosphamide clearance were reported across tumour types, marked differences in drug clearance and metabolism are observed between the current data and recently published results from a comparable study conducted by our group in a paediatric Non-Hodgkin’s Lymphoma (NHL) patient population. Data from the previous study showed significantly lower cyclophosphamide clearances and metabolite exposures than the data generated from infants recruited to the current study. These findings suggest that age may well be an important factor to take into account when considering cyclophosphamide pharmacokinetics and drug disposition in a childhood cancer setting. Additional factors which may be important to consider include the potential for induction or saturation of cyclophosphamide metabolism, which may impact on the differences between the studies. However, clearance values were determined following the first cyclophosphamide dose in both studies, therefore there is not sufficient time for auto-induction of metabolic enzymes. Furthermore, although patients were generally administered higher relative doses in the infant study (100–1500 mg/m^2^) than the NHL study (250 mg/m^2^), no differences in clearance were observed across the dose range investigated in the current study.

Although there are very limited published data available relating to cyclophosphamide pharmacokinetics in infants, a study recently carried out at St Jude Children’s Hospital in the US, indicated that younger infants exhibited higher exposures to 4-hydroxy-cyclophosphamide, an intermediate metabolite in the activation pathway, than older children [8]. While our study involved measurement of the inactive metabolites of cyclophosphamide as opposed to this intermediate activation metabolite, our findings that cyclophosphamide clearance is higher in infants, with a relative increase in metabolite production observed, are in line with these recent findings from the St. Jude group.

Pharmacogenetic variation has been shown to play a key role in determining cyclophosphamide clearance, with CYP2B6*6 arguably being the most important minor allele investigated [10,14,15,16]. In the current study a 30% reduction in cyclophosphamide clearance in the *1/*6 group (*n* = 7) was observed relative to the *1/*1 group (*n* = 18). Although not significant, this finding is consistent with the previously discussed paediatric NHL study, which showed a comparable 34% reduction in cyclophosphamide clearance in the *1/*6 genotype group [10]. In addition, these data are supported by the subsequent trend of increased cyclophosphamide AUC and decreased metabolite AUC_0–6_ for *1/*6 patients. This suggests that this decrease in cyclophosphamide clearance in the *1/*6 genotype is consistent across age groups. Indeed, when the clearance data for the two studies is combined, a significant reduction in clearance associated with the *1/*6 genotype is observed (Figure S3). In contrast, the other SNPs investigated (CYP2B6*5 and CYP2C19*2) in the current study were associated with less pronounced changes in clearance.

When investigating ontogeny of drug metabolizing enzymes, CYP2B6 is not of primary focus, likely due to its low contribution to metabolism (8%) of clinically relevant drugs. However, from a paediatric oncology perspective, cyclophosphamide is used as part of first line treatment in a variety of childhood cancers such as ALL, Ewings sarcoma, NHL, retinoblastoma and Wilms tumour. Therefore, the potential for childhood cancer patients to receive cyclophosphamide drug is high. Greater numbers of patients are required to determine significant differences in clearance due to ontogeny and genetic variation, to better understand when dose modifications should be applied.

In conclusion, whilst this study demonstrates no significant differences in cyclophosphamide clearance in patients during the first two years of life, it does highlight the large differences in dosing protocols across tumour types. Future studies should focus on establishing the best practice for cyclophosphamide administration, whether it be a low dose over several days or a higher dose on a single day. Furthermore, the study highlights clear differences in cyclophosphamide clearance in children less than two years of age as compared to older children. A more focused and expansive study looking at the full spectrum of ages of children, with a single tumour type and a standardized dosing regimen, would be desirable to determine the best dosing practices for cyclophosphamide in a paediatric setting.

## 4. Materials and Methods

### 4.1. Patients and Treatment

The study was approved by the UK Trent Multicentre Ethics Committee and registered through the appropriate clinical trial registries (REC: 06/MRE04/46; CTA: 17136/0245/001; EUDRACT: 2006-002845-36) ahead of patient recruitment. Participating centres were required to obtain local ethical approval and written informed consent from the parents of patients recruited to the study. Patients aged 0–2 years of age at diagnosis, receiving cyclophosphamide as standard treatment according to the relevant clinical trial protocol, were eligible. The various cyclophosphamide dosing regimens in place across active clinical trial treatment protocols during patient recruitment to this pharmacology study are described in Table S1. All patients were required to have a central venous catheter in place to participate in the study, which involved multiple blood draws for pharmacokinetic analysis. At the time of patient registration baseline toxicity values prior to cyclophosphamide administration were recorded. This included key hematological toxicities such as neutrophil, white blood cell (WBC), hemoglobin and platelet count.

Cyclophosphamide was administered at doses ranging from 100–1500 mg/m^2^ (5–75 mg/kg), with infusion times ranging from a slow bolus infusion to a 90 min infusion as part of the standard treatment regimen that each patient was receiving. Details of co-medications that patients were receiving on their chemotherapy regimens were recorded. Toxicity was assessed by the National Cancer Institute Common Toxicity Criteria (CTC), version 2.0, following cyclophosphamide treatment. Only toxicities of grade 3 or above were recorded. Patient status was assessed during follow up at six and 12 months after initial cyclophosphamide sampling.

### 4.2. Blood Sampling and Analysis

Blood samples for quantification of cyclophosphamide and the inactive metabolites, 4-ketocyclophosphamide (Keto), dechloroethylcyclophosphamide (DCCP) and carboxyphosphamide (CXCP) were obtained from a central line prior to drug administration and at multiple time points following the beginning of infusion, commonly 1, 2, 4, 6 and 24 h. Only inactive metabolites were measured due to the challenges associated with measuring 4-hydroxycyclophosphamide metabolite [8], where additional immediate stabilisation and analysis procedures are required to measure this metabolite. Given that these samples were taken from multiples sites across the UK, measurement of this metabolite was not feasible. All samples were taken from a different lumen to that used for drug administration following a standardized procedure, with the actual drug administration and sampling times recorded for all patients for pharmacokinetic analysis. Plasma was obtained from whole blood samples (2 mL) by centrifugation at 1200 g for 5 min at 4 °C and was frozen at −20 °C prior to analysis. Samples were sent by overnight courier, on dry ice and in an insulated container, to the Newcastle University Centre for Cancer, for analysis.

Sample analysis was carried out using a fully validated LC/MS assay as previously described, with a limit of quantification of 0.5 µg/mL for cyclophosphamide and 0.05 µg/mL for the metabolites [9,10]. Appropriate parent drug and metabolite quality control samples were included in each run and the assay exhibited within- and between-run coefficients of variation and bias <15%. Standard curves were linear between 0.5–10 µg/mL and 0.05–1.0 µg/mL for cyclophosphamide and its metabolites, respectively, with samples diluted as required for those containing higher drug concentrations.

### 4.3. Pharmacokinetic Analysis

Data generated for cyclophosphamide were analysed using a pharmacokinetic model previously published by our group using nonlinear mixed effects modelling (NONMEM version 7.2) in a childhood cancer patient population aged 3–19 years of age [10]. The first order conditional estimation method with η/ε interaction was used, together with the ADVAN3 and TRANS4 routines. A composite error model was most appropriate to describe within-subject error. An additive error model, on the logarithmic scale, was used for interindividual variability in pharmacokinetic parameters. One thousand data sets were simulated to provide visual predictive checks (VPC) using the population pharmacokinetic model. The median and 90% prediction intervals were plotted with the original data. Empirical Bayes estimates of pharmacokinetic parameters including clearance (CL), volume (V1) and area under the plasma concentration-time curve (AUC) were generated, with the covariates creatinine and CYP2B6*6 genotype due to their relationship with cyclophosphamide CL. To allow for differences in body size, population parameters were scaled to body surface area using the same allometric scaling approach as previously published [17].

For the cyclophosphamide metabolites, CXCP, DCCP, and Keto, concentrations were not included in the model for cyclophosphamide. Instead AUC values from time 0–6 h were calculated using the trapezoidal rule (WinNonLin v 8.0, Certara L.P. Pharsight, St. Louis, MO, USA), to allow comparisons with previously published data.

Pharmacokinetic data generated on both the parent drug and metabolites were compared with previous data generated using the same analytical techniques and analysed using the same population pharmacokinetic model in a population of 49 childhood cancer population aged 3–19 years [10].

### 4.4. Pharmacogenetic Analysis

Genomic DNA was obtained from whole blood samples using Qiagen QIAamp^®^ DNA Blood Maxi kits according to the manufacturer’s instructions. DNA purity and concentration were measured using a NanoDrop ND-1000 (Thermo Scientific, Rockford, USA) and stored at −20 °C prior to pharmacogenetic analysis. Genotyping was carried out for SNPs CYP2B6*4 785A > G (rs2279343), CYP2B6*5 1459C > T (rs3211371), CYP2B6*6 785A > G (rs2279343) & 516G > A (rs3745274), CYP2C19*2 681G > A (rs4244285), CYP2C19*17 806C > T (rs12248560), *GSTP1* 313A > G (rs1695), *CAR* 540C > T (rs2307424), and *PXR*-25385C > T (rs3814055) using TaqMan^®^ probes and an ABI 7500 Fast Real-Time PCR System (Applied Biosystems, Foster City, CA, USA). Allelic discrimination was performed using sequence detection software (Applied Biosystems).

### 4.5. Statistical Analysis

Differences in cyclophosphamide and metabolite exposure or clearance were assessed in terms of age, weight and genotype. Age was separated into three groups: 0–6, 7–12, and 13–24 months. To investigate the effect of weight on clearance and exposure patients were separated into two groups, above or below 10 kg. These cut off values were selected as dose reductions are typically applied to patients below 10 kg or one year of age. Differences in clearance and exposure were also compared between CYP2B6 genotype groups (SNPs *1/*1 and *1/*6). Overall differences between groups were assessed with the Mann–Whitney and Kruskal–Wallis tests (GraphPad Software, Inc., San Diego, CA, USA, version 8). Statistical significance was associated with p values < 0.05. Inter-patient variability was described using the coefficient of variation (CV), calculated using the geometric mean and standard deviation.

## 5. Conclusions

The findings from the current study highlight marked differences in cyclophosphamide dosing protocols across tumour types and clear differences in cyclophosphamide clearance in children less than two years of age as compared to older children.

## Figures and Tables

**Table 1:** Patient characteristics.

Characteristic		No.
Evaluable patients		25
Age (months)		
	0–6	4
	7–12	10
	13–24	11
Sex		
	Male	10
	Female	15
BW (kg)		
	Median	9.4
	Range	5.5–16.1
BSA (m^2^)		
	Median	0.46
	Range	0.32–0.71
Tumour Type		
	ALL	11
	AML	1
	Astrocytoma	1
	Ependymoma	1
	Neuroblastoma	7
	PNET supratentorial	1
	Posterior fossa ATRT	1
	Rhabdoid-Kidney	1
	Rhabdomyosarcoma	1

BW: body weight; BSA: body surface area; ALL: acute lymphoblastic leukaemia; AML: acute myeloid leukaemia; PNET: primitive neuroectodermal tumour; ATRT: atypical teratoid rhabdoid tumour.

**Table 2:** Summary of average cyclophosphamide pharmacokinetic parameters generated for the infants on the study.

Parameter	Geometric Mean	Range	CV%
AUC (mg/mL.min)	12.6	2.03–37.74	62
AUC/D ((mg/mL.min)/dose)	0.045	0.0013–0.1915	62
CL (mL/min)	22.1	5.22–74.46	46
CL (mL/min/m^2^)	46.6	9.32–151.95	41
V1 (L/m^2^)	9.7	4.88–16.68	25

AUC: area under the plasma concentration-time curve; D: dose; CL: clearance; V1: volume.

## Data Availability

The data presented in the current study are available from the corresponding author upon reasonable request.

## Supplementary Materials

The following are available online at https://www.mdpi.com/1424-8247/14/3/272/s1, Figure S1: Diagnostic plots from the population pharmacokinetic model showing (A) individual predicted concentrations vs. observed concentrations, (B) population predicted concentrations vs. observed concentrations, (C) time vs. weighted residuals and (D) population predicted concentrations vs. weighted residuals. Panel (E) shows the visual predictive check (VPC) for the final model; Figure S2: The effect of (A) age, (B) CYP2B6 genotype and (C) weight on cyclophosphamide AUC normalised to dose and (D) weight not normalised to dose. The error bars represent standard deviation, the black dashed line indicates the geometric mean cyclophosphamide normalised AUC of 0.045 (mg/mL.min)/dose and the blue dashed line indicates the geometric mean cyclophosphamide AUC of 12.6 mg/mL.min; Figure S3: The effect of genotype on clearance using data generated from two studies in childhood cancer patient populations. Error bars represent standard deviation, *** indicates a significance level of *p* < 0.0001; Figure S4: The effect of tumour type on (A) clearance (mL/min/m^2^) and (B) AUC (mg/mL.min). The tumour type “Other” refers to the remaining tumour types in the study. The error bars represent standard deviation, the blue dashed line indicates the geometric mean Clearance (46.6 mL/min/m^2^) and AUC (12.6 mg/mL.min); Table S1: Summary of dosing regimens for patients recruited to the study; Table S2: Patient toxicities reported following administration of cyclophosphamide (only toxicities of grade 3 or above were recorded) (*n* = 25); Table S3: Pharmacokinetic parameter final estimates.
