# Supplementary material for: Pharmacokinetics and Pharmacogenetics of Cyclophosphamide in a Neonate and Infant Childhood Cancer Patient Population
Source: Pharmaceuticals (Basel). 2021 Mar 16;14(3):272. doi: 10.3390/ph14030272 (PMC8002238; doi:10.3390/ph14030272)

Supplemental files

Table S1 Summary of dosing regimens for patients recruited to the study.

Dosing Regimen	Dose Reduction	Number of Patients
500 mg/m^2/day x 2	6-12 months–75% dose; <6 months–66% dose	3
1 g/m^2/day	7-≤12 months–75% dose; ≤ 6 months–66% dose	11
4 g/m^2/course		4
50 mg/kg/day		2
5 mg/kg/day x 5		2
10 mg/kg/day x 5		3

Table S2 Patient toxicities reported following administration of cyclophosphamide (only toxicities of grade 3 or above were recorded) (*n*=25).

**Toxicity**	**Number of patients** (%)		
	**Grade 3**	**Grade 4**	**Grade 3/4**
Neutrophil	4 (16)	8 (32)	12 (48)
Haemoglobin	12 (48)	0 (0)	12 (48)
White Blood Cell	6 (24)	4 (16)	10 (40)
Fever	0 (0)	4 (16)	4 (16)
Diarrhoea	0 (0)	2 (8)	2 (8)
Infection	0 (0)	4 (16)	4 (16)
Platelets	3 (12)	0 (0)	3 (12)
Stomatitis	0 (0)	1 (4)	1 (4)
Central Neurotoxicity	1 (4)	0 (0)	1 (4)
Peripheral Neurotoxicity	1 (4)	0 (0)	1 (4)
SGOT/SGPT	0 (0)	1 (4)	1 (4)
Other	1 (4)	0 (0)	1 (4)

Toxicity was assessed by the National Cancer Institute Common Toxicity Criteria (CTC), version 2.0

Table S3 Pharmacokinetic parameter final estimates

Parameter	Estimate	CI 95%	IIV (CV%)	η-Shrinkage
Clearance (L/min)	0.0225	[0.0197–0.0252]	46	15%
Volume 1 (L)	4.6	[4.3–4.9]	20	17%
Q (L/min)	0.128	[0.113–0.142]	56	52%
Volume 2 (L)	1.72	[1.66–1.79]	15	99%
**Residual error**				**ε-shrinkage**
Residual error ε-shrinkage				32.73%

CI 95%: confidence interval 95%; IIV: inter-individual variability

Figure S1 Diagnostic plots from the population pharmacokinetic model showing (**A**) individual predicted concentrations vs. observed concentrations, (**B**) population predicted concentrations vs. observed concentrations, (**C**) time vs. weighted residuals and (**D**) population predicted concentrations vs. weighted residuals. Panel E) shows the visual predictive check (VPC) for the final model.

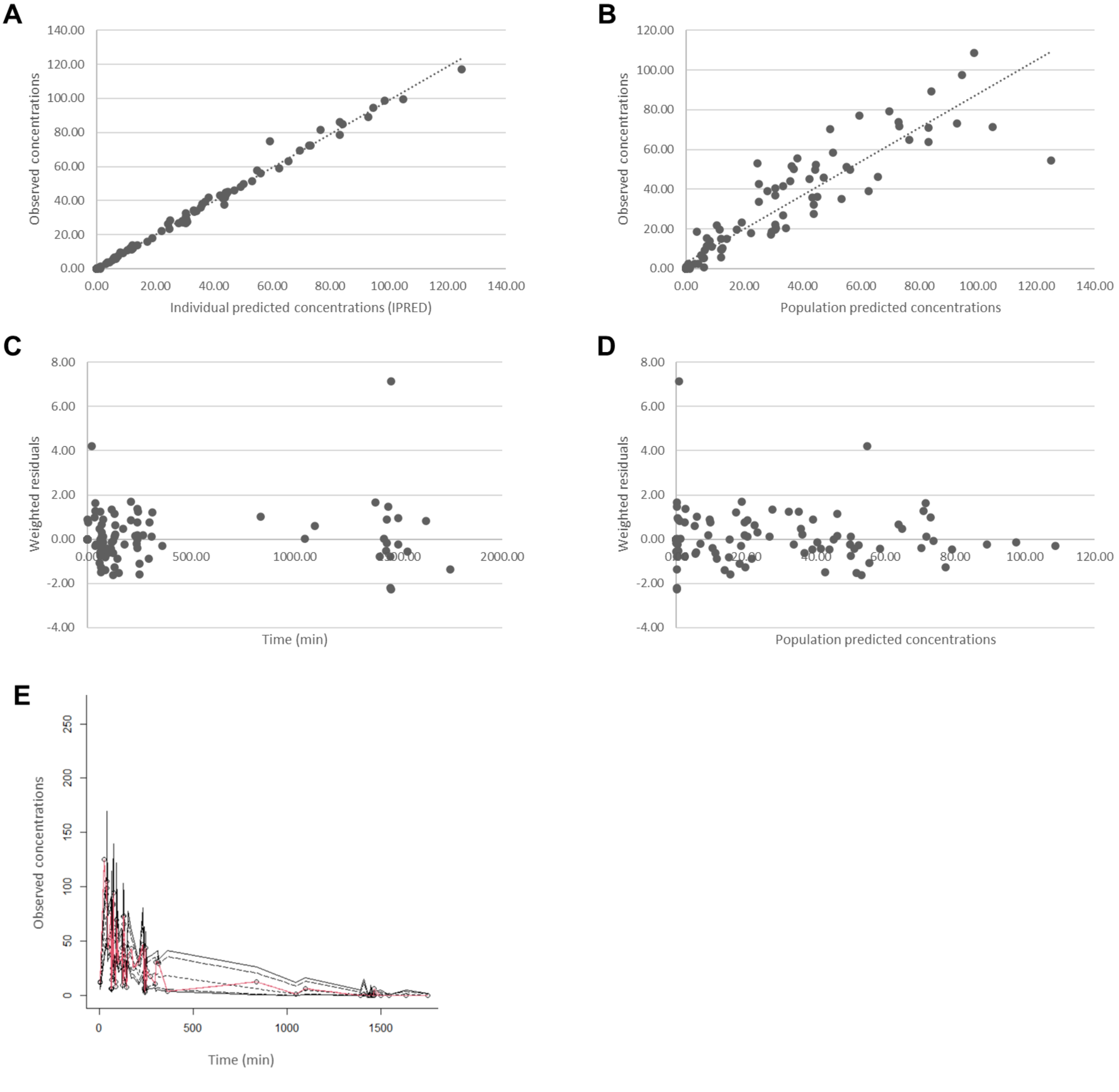

Figure S2 The effect of (**A**) age, (**B**) CYP2B6 genotype and (**C**) weight on cyclophosphamide AUC normalised to dose and (**D**) weight not normalised to dose. The error bars represent standard deviation, the black dashed line indicates the geometric mean cyclophosphamide normalised AUC of 0.045 (mg/mL.min)/dose and the blue dashed line indicates the geometric mean cyclophosphamide AUC of 12.6 mg/mL.min.

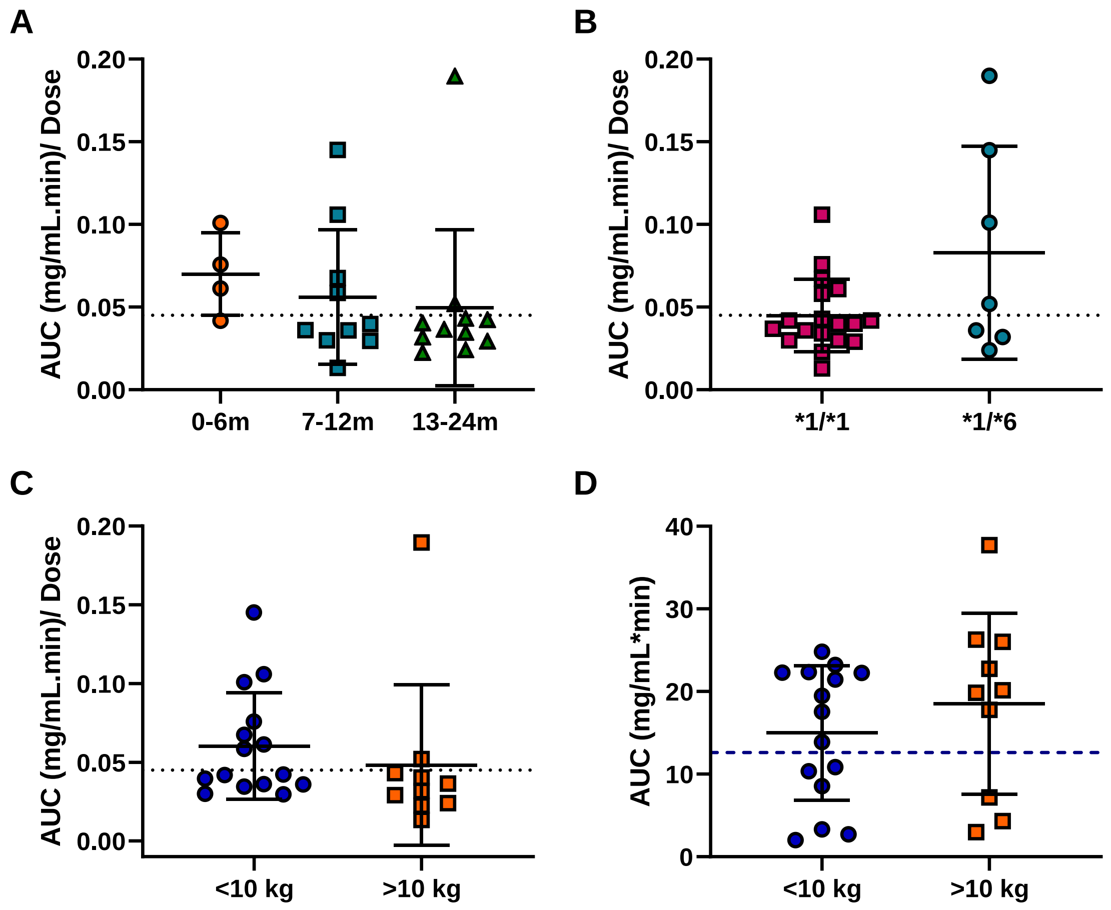

Figure S3 The effect of genotype on clearance using data generated from two studies in childhood cancer patient populations. Error bars represent standard deviation, *** indicates a significance level of *P* <0.0001.

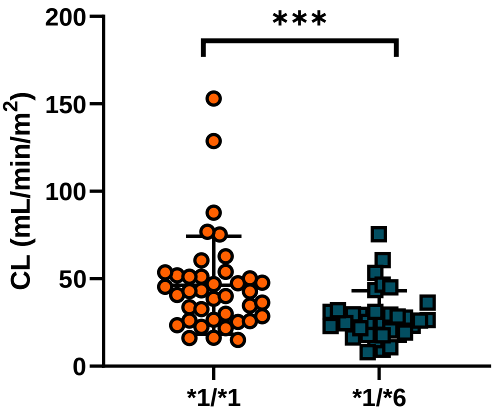

Figure S4 The effect of tumour type on (**A**) clearance (mL/min/m^2) and (**B**) AUC (mg/mL.min). The tumour type "Other" refers to the remaining tumour types in the study. The error bars represent standard deviation, the blue dashed line indicates the geometric mean Clearance (46.6 mL/min/m^2) and AUC (12.6 mg/mL.min).

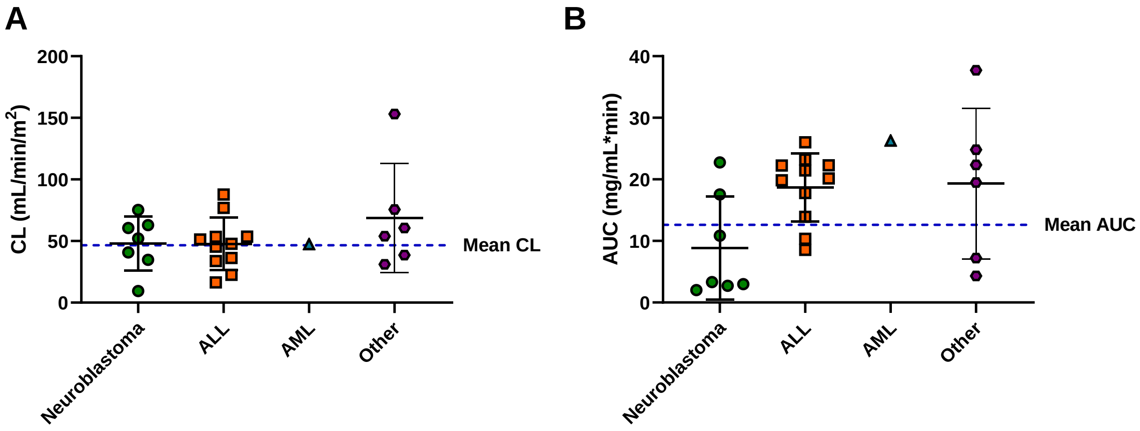

Supplement: Supplementary file 1 [file pharmaceuticals-14-00272-s001.pdf]
